# Weight gain prevention in young adults: design of the study of novel approaches to weight gain prevention (SNAP) randomized controlled trial

**DOI:** 10.1186/1471-2458-13-300

**Published:** 2013-04-04

**Authors:** Rena R Wing, Deborah Tate, Mark Espeland, Amy Gorin, Jessica Gokee LaRose, Erica Ferguson Robichaud, Karen Erickson, Letitia Perdue, Judy Bahnson, Cora E Lewis

**Affiliations:** 1 The Miriam Hospital/Brown University, 196 Richmond Street, Providence, RI 02903, USA

**Keywords:** Weight gain prevention, Young adults, Obesity, Self-regulation, Self-weighing

## Abstract

**Background:**

Weight gain during young adulthood is common and is associated with increased cardiovascular risk. Preventing this weight gain from occurring may be critical to improving long-term health. Few studies have focused on weight gain prevention, and these studies have had limited success. SNAP (Study of Novel Approaches to Weight Gain Prevention) is an NIH-funded randomized clinical trial examining the efficacy of two novel self-regulation approaches to weight gain prevention in young adults compared to a minimal treatment control. The interventions focus on either small, consistent changes in eating and exercise behaviors, or larger, periodic changes to buffer against expected weight gains.

**Methods/Design:**

SNAP targets recruitment of six hundred young adults (18–35 years) with a body mass index between 21.0-30.0 kg/m^2^, who will be randomly assigned with equal probability to: (1) minimal intervention control; (2) self-regulation with Small Changes; or (3) self-regulation with Large Changes. Both interventions receive 8 weekly face-to-face group sessions, followed by 2 monthly sessions, with two 4-week refresher courses in each of subsequent years. Participants are instructed to report weight via web at least monthly thereafter, and receive monthly email feedback. Participants in Small Changes are taught to make small daily changes (~100 calorie changes) in how much or what they eat and to accumulate 2000 additional steps per day. Participants in Large Changes are taught to create a weight loss buffer of 5–10 pounds once per year to protect against anticipated weight gains. Both groups are encouraged to self-weigh daily and taught a self-regulation color zone system that specifies action depending on weight gain prevention success. Individualized treatment contact is offered to participants who report weight gains. Participants are assessed at baseline, 4 months, and then annually. The primary outcome is weight gain over an average of 3 years of follow-up; secondary outcomes include diet and physical activity behaviors, psychosocial measures, and cardiovascular disease risk factors.

**Discussion:**

SNAP is unique in its focus on weight gain prevention in young adulthood. The trial will provide important information about whether either or both of these novel interventions are effective in preventing weight gain.

**Trial registration:**

ClinicalTrials.gov, NCT01183689

## Background

Young adults, ages 20–35, experience the greatest rate of weight gain, averaging 1 to 2 pounds per year [1,2]. Over time, this weight gain is associated with a worsening in cardiovascular risk factors and an increase in the prevalence of metabolic syndrome [3,4]. To date, there have been few large trials designed to test ways to prevent weight gain in this age group, and the results have been disappointing. The present paper describes two new approaches to weight gain prevention and the design of a multi-site randomized controlled clinical trial that is underway to examine the efficacy of these approaches.

### Weight gain in young adults

A number of studies have documented significant weight gain in young adults. In the Coronary Artery Risk Development in Young Adults (CARDIA) study, individuals aged 18–30 gained approximately 15 kg over 15 years or 1 kg/year [1]. A study of over 8500 young women, aged 18–23, found that 41% gained more than 5% over their baseline weight over 4 years [5]. Getting married, pregnancies, and entering the work force have all been related to weight gain in this age group [5,6]. Moreover, the weight gained by young adults has adverse health consequences. In the CARDIA study, only 16.3% of young adults maintained a stable BMI over 15 years of follow-up, but those individuals who remained weight stable had essentially unchanged levels of all of the components of the metabolic syndrome, regardless of their initial body mass index, age, race, or gender. In contrast, those who gained weight had worsening in cardiovascular risk factors and increased prevalence of the metabolic syndrome [1,7]. Weight gained during young adulthood has also been associated with increased risk of coronary heart disease events [8] and a variety of other diseases, including postmenopausal breast cancer, kidney stones, gout, hypertension and type 2 diabetes [9-12]. These studies suggest that preventing weight gain in young adults would decrease the risk of cardiovascular disease and improve overall health.

### Prior trials for weight gain prevention in young adults

To date, there have been few randomized trials testing interventions specifically designed to target weight gain prevention and the two largest, longest trials had limited effects. In Pound Of Prevention (POP) [13], weight gain over 3 years was examined in a no-contact control group compared with a group given education through monthly newsletters and a group given the same education plus incentives for participation. None of the interventions were successful in reducing the average magnitude of weight gained over 3 years (1.8 kg in control; 1.6 kg in education and 1.5 kg in education plus incentive). The other large prevention trial by Levine et al. [14] compared an in person approach, a correspondence program and a no treatment control in a sample of 284 female participants, aged 25 – 44 with a BMI of 21 – 30 kg/m^2^ followed for 3 years. Mean weight changes over 3 years did not differ significantly between conditions (+0.7 kg, +0.3 kg, and −0.6 kg for the control, correspondence, and in-person conditions, respectively). Clearly new approaches to weight gain prevention for young adults are needed. Recognizing this need, National Heart, Lung, and Blood (NHLBI) has funded several trials on this topic and developed the Early Adult Reduction of weight through LifestYle intervention (EARLY) consortium (Lytle L, Svetkey LP, Patrick K, Belle SH, Fernandez ID, Jakicic J, Johnson KC, Olson C, Tate DF, Wing RR: The EARLY trials: a consortium of studies targeting weight control in young adults, submitted).

### Novel interventions for weight gain prevention

One way to prevent weight gain is to engage in a process of self-regulation of behavior, an approach we tested successfully in the prevention of weight regain [15] but has not yet been tested for weight gain prevention. Self-regulation involves having a goal, having access to information about whether the goal is being achieved, and if not, taking steps to restore equilibrium. For example, applying self-regulation to type 1 diabetes, an individual must be knowledgeable about the level of blood glucose they are trying to achieve, they must monitor their glucose to see if there are discrepancies between their goal and their actual blood sugar, and then if there are discrepancies, they must adjust their diet, exercise, or insulin dose to reduce the discrepancy. Within the area of weight gain prevention, the individual has a goal of maintaining their current weight. Information about deviations from this goal is best provided by frequent self-weighing. Although scales are not perfect, they provide more accurate and immediate feedback than other indicators such as noticing if one’s clothes are too tight. If discrepancies are noted, the individual must change their behavior to reduce the discrepancies. It remains unclear, however, what type of behavior changes the individual should make to reduce this discrepancy. Two different approaches have been suggested—a “Small Change” and a “Large Change” approach.

Currently the message being given to the public is that daily small changes in eating and exercise behavior will prevent weight gain. The America on the Move Foundation (https://aom3.americaonthemove.org/) encourages Americans to take small, simple lifestyle changes - versus dramatic changes - to ensure effective long-term weight control. This message is based on the fact that the average weight gain with aging is about 1 kg per year; therefore, a decrease of 10–15 kcal/day (or 20–30 kcal/day based on the estimated 50% energy cost to storing excess energy), should be sufficient to prevent weight gain. Using these calculations, Hill and colleagues [16] have suggested that if we could modify energy imbalance by 100 kcal/day through small changes in eating and/or physical activity, we could prevent weight gain in 90% of the U.S. adult population. Behavioral theory also suggests that small changes (i.e. gradual shaping of new behaviors with small incremental changes toward a goal) should be easier to initiate and maintain than larger behavior changes since they represent less drastic modifications in behavior [17]. Although several recent studies have provided empirical support for this approach, these studies have been short in duration (13–24 weeks) and the recommended changes (e.g. consuming cereal for 2 meals per day) could be considered relatively substantial in nature, as opposed to representing small and easily integrated changes based on one’s current lifestyle, as suggested by the AOM campaign.

Another approach to weight gain prevention is to produce initial weight loss as a buffer against the expected weight gain, which we refer to as the “Large Change” approach. There is stronger empirical evidence for this approach, coming from the Women’s Healthy Lifestyle Project (WHLP), the only study that has actually succeeded in preventing weight gain and the worsening in CVD risk factors over a period of 5 years [18]. In this study conducted with women (BMI of 20 to 34) during the menopausal transition, the intervention group was encouraged to lose 5–15 pounds as a means of counteracting the weight gains that is expected with aging. The intervention group lost a mean of 0.09 kg over the 5-year intervention whereas the assessment only group gained 2.4 kg. The intervention also reduced the worsening in cholesterol during this time period. A large behavior change approach is also supported by a secondary analysis of data from the POP study. Jeffery, McGuire and French [19] found that although weight loss was not targeted, 9.3% of their study population lost >5% (mean= 6.4 kg) in Year 1; these individuals were the only group that was below baseline at year 3 (−2.6 kg). Similarly in the Levine [14] study, 70% of the participants who lost > 2.3 kg from baseline to 1 year were still below their baseline weight at year 3; among those who lost 0.9–2.3 kg at 1 year, 60% were still below baseline at year 3. However, among those who were weight stable from baseline to year 1 (± 0.9 kg) only 35% were still below baseline at 3 years. To date, there have been no prior trials comparing the small and large changes approach and examining their efficacy for preventing weight gain in young adults.

### Study aims

The Study of Novel Approaches to Weight Gain Prevention (SNAP) is a two site, randomized clinical trial funded by the National Heart Lung and Blood Institute. The two clinical sites are at The Miriam Hospital (R. Wing, PI) and The University of North Carolina at Chapel Hill (D. Tate, PI). The Coordinating Center is at Wake Forest School of Medicine (M. Espeland, PI). SNAP is comparing the efficacy of self-regulation plus small behavior changes intervention, a self-regulation plus large behavior changes intervention, and a minimal treatment control condition in preventing weight gain in 600 young adults age 18 – 35 over an average planned follow-up of 3 years.

The primary hypothesis is that the magnitude of weight gain across 3 years will differ among the three groups. Specific *a priori* hypotheses are that the magnitude of weight gain across the 3 years will be lowest in the self-regulation plus large behavior changes intervention, followed by the self-regulation plus small behavior changes intervention, and greatest in the control condition.

Secondary aims compare the 3 conditions on mean weight gain at 2 years, on the proportion who gain <1 pound or >1 pound at 3 years, and on the proportion who become obese at 3 years. The three groups are also compared on changes in behavior (e.g. diet, physical activity, disordered eating behaviors, use of healthy and unhealthy weight control practices), psychosocial measures (restraint, depression), and changes in CVD risk factors (including blood pressure, lipids, insulin sensitivity, and waist circumference). The study will also examine demographic and psychological variables that may predict weight change over the average follow-up of 3 years and/or moderate the effects of the interventions, including initial BMI, ethnicity, age, scores on the Eating Inventory, and treatment preference and examine potential mediators of the effect of the interventions, including changes in diet, physical activity, restraint, self efficacy and frequency of self-weighing.

## Methods

### Study design

SNAP targets the enrollment of 600 participants, who are randomly assigned with equal probability to 1 of 3 treatment groups, stratified by clinical center:

1. Self-guided behavior changes (Control)

2. Self-Regulation Plus Small Behavior Changes

3. Self-Regulation Plus Large Behavior Changes

Assessments are completed at baseline, 4, 12, 24, 36 and 48 months. Since enrollment is staggered, all participants are scheduled to complete the 24-month follow-up, 80% are scheduled to complete 36 months and 20% are scheduled to complete the 48-month assessments. The study was approved by the Institutional Review Boards at Lifespan-The Miriam Hospital, The University of North Carolina at Chapel Hill and Wake Forest University Health Sciences.

### Eligibility

The recruitment goal is to randomize 600 participants, with at least 25% men and 25% from racial/ethnic minority groups. Table 1 describes the eligibility and exclusion criteria for this trial. As indicated, all participants must be 18–35 years old. This age group was selected because young adults have the greatest risk of weight gain over time. The BMI range of 21-30 kg/m^2^ was selected since weight gain prevention seems an appropriate message for these individuals. For individuals with a BMI of <21 kg/m^2^, changes in behavior resulting in weight losses could result in BMIs outside the normal weight category (i.e. less than 18.5). We selected a BMI of 30 kg/m^2^ as our upper cutoff since individuals with a BMI >30 kg/m^2^ are considered obese, and weight loss (rather than weight gain prevention) is typically recommended for these individuals [20]. The exclusion criteria were developed to maximize the safety of the intervention and minimize the likelihood that a participant would complete the full trial while also taking into account the generalizability of the findings. Exclusion criteria include: 10 pound weight loss in the past six months, bariatric surgery, hospitalization for depression or psychiatric disorder, history of bipolar disorder, manic depression or schizophrenia, past diagnosis or treatment for anorexia or bulimia nervosa, past diagnosis or current symptoms of alcohol or substance abuse, or currently pregnant or nursing within the past 6 months or planning to become pregnant within the next 6 months.

**Table 1:** Inclusion and exclusion criteria for SNAP

Inclusion criteria	
	• BMI 21 – 30 kg/m^2^
	• 18 – 35 years of age
	• Willing to be randomized to any of the three conditions
Exclusion criteria	
	• Untreated hypertension, hyperlipidemia, or type 2 diabetes, unless permission is provided by their health care provider
	• Unable to walk for physical activity
	• Report of any of the following health problems: heart condition, chest pain during periods of activity or rest, loss of consciousness, diabetes treated with insulin or medications that may cause hypoglycemia, active tuberculosis, HIV, acromegaly, Cushing’s syndrome, chronic hepatitis B or C, inflammatory bowel disease requiring treatment within the past year, thyroid disease, renal disease, liver disease, hospitalization for asthma in the past year, or cancer within the past 5 years (except for non-melanoma skin cancers or early stage cervical cancer) or chronic use of steroid medication
	• Past diagnosis of or treatment for anorexia nervosa or bulimia nervosa or meet criteria for anorexia or bulimia nervosa during screening
	• Past diagnosis of or current symptoms of alcohol/substance dependence
	• Currently pregnant, trying to get pregnant, pregnant within last 6 months
	• History of schizophrenia, manic depression, or bipolar disorder
	• Hospitalization for depression or other psychiatric disorder in past 12 months
	• Lost and maintained 10 pounds or more within the past 6 months, participating in a weight loss program, taking weight loss medication, or have had surgery for weight loss
	• Participation in another weight loss or physical activity study
	• Another member of the household is a participant or staff member on this trial
	• Reason to suspect that the participant would not adhere to the study intervention or assessment schedule
	• Not able to speak and understand English
	• Residence or place of work further than 50 miles from the intervention site
	• Perceived inability to attend the 2 year data collection visit
	• No Internet access on a regular basis

### Recruitment

**Figure 1:**
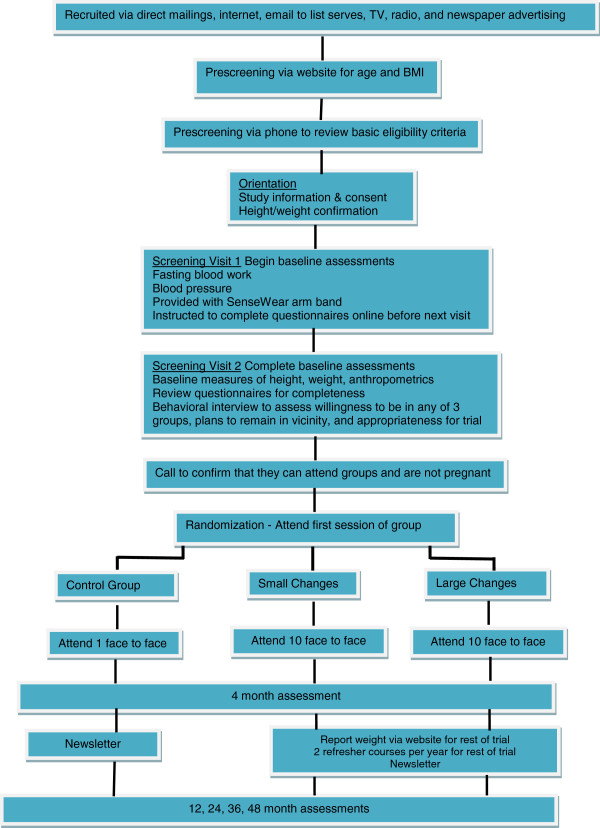
Overview of SNAP study.

Interested participants are directed to the SNAP recruitment website where they are provided with basic information about the study and asked to complete an initial eligibility form reporting their age, sex, height and weight (for determination of BMI). Subsequently those who appear eligible are further screened by phone and then, if still eligible, asked to attend an orientation meeting, complete informed consent and are then scheduled for baseline screening visits. The flow chart for these stages of recruitment is shown in Figure 1.

### Randomization

Randomization follows a simple, non-adaptive variable-block length algorithm, which is stratified by clinical site and by gender and ethnicity (Non-Hispanic white versus other race/ethnic groups).

### Self-guided behavior changes (Control condition)

Participants in the self-guided condition receive one face-to-face group session in which information about behaviors associated with weight gain in young adults and the health consequences of weight gain are presented. Self-weighing is introduced as a preventive strategy, both the small changes and large changes approaches are described, and 2–3 internet resources consistent with each approach are provided. In addition, participants receive an overview of the principles of both the Large and Small Changes approaches and are encouraged to select whichever approach they feel will work best for them and use it to prevent weight gain over the course of the study. Participants have access to a study website where quarterly newsletters are posted with information about weight gain prevention and healthy eating and physical activity strategies, as well as links to the internet resources mentioned above, but no active assistance is provided in implementing these approaches.

### Active interventions: common components

The two active interventions tested in SNAP are both based on self-regulation theory and share an emphasis on daily self-weighing; they also have the same frequency of interventionist contact (see Table 2), provide the same basic information about healthy eating and physical activity, and teach participants identical behavior modification skills to facilitate the prescribed behavior changes (small or large). The same interventionists, who have master’s level backgrounds in nutrition, exercise physiology or psychology, and previous behavioral weight control experience, lead groups for both intervention arms.

**Table 2:** Schedule of intervention contact

**Time frame**	**Meetings**	**Reporting weight**
Year 1		
Months 1-2	Weekly face-to-face (8 total)	None*
Months 3-4	Monthly face-to-face (2 total)	Weekly
Months 5-12	None	Monthly
Year 2	Two annual 4 week refreshers: offered in different formats (face-to-face; online; email)	Monthly
Year 3	Two annual 4 week refreshers: offered in different formats (face-to-face; online; email)	Monthly

*No reporting is needed since participants are weighed at weekly meetings.

#### Intervention contact

The core content of both intervention conditions is delivered in 8 weekly group sessions, followed by 2 monthly groups (e.g., initial 10 session program delivered over 16 weeks). Following initial treatment, monthly contact from the SNAP team is maintained primarily via automated reminder emails for weight submission, and a monthly email with feedback on weight zone and recommended behavioral strategies. Additional modalities are used depending on participant zone status, including monthly postal mail for sending token reinforcers, and phone, email or in-person contact if requested by participants who have gained above baseline. Two annual refresher campaigns (4 weeks in duration) are offered using in-person sessions, email or other Internet modalities to offer different approaches to appeal to different participants. While refresher formats may vary over time, the identical format (with content/goals appropriate for the treatment condition) is offered to Small and Large Changes.

#### Self-regulation

The goal of both interventions is to prevent weight gain through the self-regulation of eating and exercise behaviors, a model that was used successfully in STOP Regain [15]. Participants are given scales for home use and are taught core self-regulation skills, including: a) to weigh themselves daily; b) to detect small changes in weight as soon as they occur; c) to implement problem solving and behavioral strategies to deal with these changes; d) to evaluate the success of these strategies; and e) to provide self-reinforcement for successful weight maintenance or to make changes in their behavior if gains occur. To help participants detect small changes in their weight and to guide appropriate actions, they are taught to use a red, yellow, and green weight monitoring system. Based on their preference, participants use either a web-based or Short Message Service (SMS) to report their weight at least monthly; based on their reported weight, they receive immediate automated feedback as to their color zone for the week (see color zone descriptions below) and are instructed to practice either reinforcing their own success or taking the corrective appropriate action. Participants also receive monthly email tip sheets corresponding to their color zone from a study interventionist. The color zones for both interventions are identical. For participants who lost weight during the initial 8 week intervention, the green zone (Go!) is established based on their new weight allowing a minimal amount of regain. For participants ending the initial 8 week intervention at or above their starting weight, the green zone is established as less than 1 lb below their starting weight. These participants are encouraged via email to reinforce themselves for their success and receive small “green” tokens via postal mail from the study staff (e.g. green tea, dollar bill) on a preset intermittent schedule of approximately once per month to teach the benefits of reinforcement. A participant is considered to be in the “yellow zone (Caution!) if his/her current weight is above their green zone weight but at or below their starting weight. These participants are encouraged to return to self-monitoring of diet and activity (consistent with their treatment condition), to identify behavior changes that may be causing the weight gain, and to use problem solving and behavior change strategies to reverse these changes. Finally, a participant is considered to be in the “red zone” (Stop!) if his/her current weight is above their starting weight. If this occurs, behavior changes consistent with the participant’s treatment condition are prescribed. These participants are also offered up to two 20-minute individual sessions with an interventionist, either in-person or via phone or email, that focus on motivation, problem solving, and a review of techniques consistent with the participant’s treatment condition. A tip sheet encouraging behavior change is emailed to all participants in the yellow or red zones on a monthly basis with quarterly phone calls to those who have not submitted their weight or are in the red zone.

#### Basic information about self-weighing, healthy eating and physical activity

Participants in both Small Changes and Large Changes are taught about energy balance, the importance of daily weighing as an indicator of energy balance, how body weight relates to energy intake and expenditure, appropriate portion sizes, calories in protein, fat, and carbohydrates, and basic nutrition skills such as label reading. A heart healthy diet, with a low intake of saturated fat and trans-fats and high intake of fruits, vegetables and whole grains, is encouraged. Fast food consumption [5,21], alcohol consumption, and sweetened beverages [5,22] are discussed as major contributors to weight gain in young adults. General information about calories burned in different types of activity is presented to both intervention groups and the importance of both programmed and lifestyle physical activity is stressed. The interventions also seek to decrease time spent in sedentary activity since this has also been related to weight change in young adults [5]. This information is delivered in group sessions and included in quarterly newsletters sent via mail, email and available on the study website.

#### Behavior modification skills

In addition to education about healthy eating and physical activity, participants in both groups receive instruction in core cognitive and behavioral skills such as self-monitoring, stimulus control, problem solving, social support and assertiveness training, goal setting, and cognitive change strategies to help them implement their small or large behavior changes [23-27].

### Active interventions: differences between the two active interventions

The differences in the two active interventions are summarized in Table 3 and described below.

**Table 3:** Differences between two active intervention conditions in SNAP

**Key intervention concepts**	**Small changes**	**Large changes**
Dietary changes recommended for maintaining weight (green zone)	Instructed to make one small change in diet every day (roughly equivalent to 100 calories)	Start with 1200–1800 kcal/day diet to create weight loss buffer in first 8 weeks. After buffer created, gradually increase caloric intake until maintaining weight loss, but continue to consume low calorie, low fat healthy diet
Physical activity changes recommended for maintaining weight (green zone)	Given pedometers and instructed to increase steps by 2000 steps per day over baseline levels and maintain this level	Instructed to exercise to 250 min/week (50 min/day on 5 days/week) throughout the entire program.
Self-monitoring of behavior changes	During first 16 weeks and during refresher courses, record number of steps per day and check off whether or not a small change in diet was made every day. Self-monitor weight daily throughout the entire program.	Self-monitor food intake (calories and fat grams) for first 16 weeks of the program, throughout the refresher course, and if they experience weight regain. Self-monitor weight daily throughout the entire program.
What to do if regain 1 pound or more above the weight they achieve at the end of the initial 8 week program and enter yellow zone, but remain at or below baseline	Taught to resume self-monitoring of steps and small changes to diet. Use problem-solving skills, with an emphasis on changing surrounding environment to support small changes.	Taught to resume self-monitoring of food intake for several days to help identify problem areas and get back on track. Use problem-solving skills, with an emphasis on changing surrounding environment to support large changes.
What to do if exceed baseline or starting weight (red zone)	Instructed to implement additional small change(s) in both eating and exercise (e.g. make at least 1 small change at each meal, each day and increase steps by 3000 over baseline level).	Instructed to reinitiate large changes – return to 1200–1800 kcal/day diet, continue 250 min/week of activity, and self-monitor intake and activity until they are back in the green zone.

#### Self-regulation plus small behavior changes

The Self-Regulation Plus Small Behavior Changes Intervention focuses on making small changes in diet and physical activity on a daily basis to prevent weight gain and perhaps even lose some weight. The initial program helps participants identify and practice small changes which they will continue to implement on a consistent, permanent basis to prevent weight gain (see Table 4 for specific lesson topics).

**Table 4:** Lesson topics for the initial 8 week interventions in SNAP

**Week**	**Small changes**	**Large changes**
1	Small changes, big rewards: a self-regulation approach to controlling your weight	Lose a little now to get big rewards later: a self-regulation approach to controlling your weight
2	Moving more: increasing your physical activity	Healthy diet
3	Small changes in your diet: decreasing how much you eat	Moving more: taking time for physical activity
4	Making small changes in your diet that will work for you: changing what you eat	Cues in your environment
5	Small steps for big benefits	Eating out
6	The slippery slope of eating out	Problem solving
7	Liquid calories: what’s in your glass	Maintaining an exercise routine
8	Putting it all together	Putting it all together

**Diet** Participants are taught to identify small dietary changes of approximately 100 calories that they can make each day. Specific areas of focus include reducing the amount of foods consumed (e.g. leaving 3–4 bites of food on their plate), modifying the types of food they eat (e.g. eating grilled rather than fried foods), making small changes when eating out, and reducing liquid calories. For each type of dietary change, the group brainstorms possible small changes and a list of suggestions is provided to participants. Changes of approximately 100 calories are encouraged, however, it is recognized that the calorie value of each specific dietary change will vary. The general concept is that these are small, manageable changes that will produce small reductions in overall intake and can easily be made on a daily basis and maintained over time.

**Exercise** At the start of the program, participants are given a pedometer and asked to record their current number of steps for the first week; the average number of steps across the initial week represents their baseline level. They are then given the goal of increasing their daily steps by 2000 steps per day over this baseline level using small changes in lifestyle activities (e.g. walking the dog, mowing the lawn, increasing walking for transport). Additional small changes can be implemented if a selected strategy is not sufficient and in later weeks of the program adding additional minutes to their current regimen of structured exercise (e.g. bike riding for 10 minutes more) is discussed (step conversion charts are provided).

**Self-monitoring** Participants are given a monthly chart to record their daily weight, steps, and whether they made a small change in their diet during that day. The Small Changes group completes this chart daily throughout the first 16 weeks of the program and during refresher courses; these self-monitoring records are reviewed by interventionists and brief feedback provided. Participants are instructed to continue to record their weight daily throughout the entire trial.

**Maintenance** After the initial 16 week program, participants are instructed to continue to weigh daily and to make one change in diet each day and one or more changes in activity to maintain their daily step goal (i.e., baseline + 2000). If weight gain occurs at any time over the three years (i.e. enter the yellow zone), participants are taught to immediately return to self-monitoring of diet small changes and to use their pedometer and the monthly chart to confirm that they are still making daily small changes in diet and achieving their step goal. They are taught to problem solve about the causes of the weight gain. If weight regain continues or participants enter the red zone, they are taught to add additional small changes in both diet and activity to tip the energy balance (two small activity (about 3000 steps over baseline) and three small diet changes (one at each meal) equals approximately a 400–500 calorie deficit which should produce a 1 pound/week weight loss). Finally, when in the red zone, participants are encouraged to contact the program staff for additional counseling and guidance.

**Refresher courses** At each refresher course or email campaign, members of this group are asked to again monitor their steps and check off whether they are making a small change in diet each day. Participants who have experienced weight gains are encouraged to increase to three small changes in eating each day and to a step level of 3000 steps over baseline. In addition, the refresher program includes a physical activity or a nutrition activity that is fun for participants and helps motivate attendance and weight control.

#### Self-regulation plus large behavior changes group

The focus of this intervention group is on making periodic large changes in diet and physical activity, with the goal of losing 5–10 pounds to buffer against the weight gain that often occurs during young adulthood. Recognizing that it is challenging for young adults to pay close attention to diet and exercise at all times, this group is encouraged to spend a few weeks each year really focusing on diet and exercise to produce a 5–10 pound weight loss, and then throughout the rest of the year, focus on weighing themselves regularly and maintaining healthy eating habits and high physical activity levels to prevent weight regain (see Table 4 for specific lesson topics).

**Diet** Individuals with a BMI of 21–24.9 kg/m^2^ are encouraged to lose 5 pounds; those with a BMI of 25–30 kg/m^2^ are encouraged to lose 10 pounds. To produce these weight losses, participants in the Large Changes group are instructed to consume either 1200–1500 or 1500–1800 calories per day, with <30% of calories from fat. The specific calorie range is individualized based on baseline weight and physical activity. To stay within their recommended calorie range, participants are taught about calorie balance and about the calorie content of different types of foods. They are given a variety of low calorie low fat eating plans that they can use since this type of structure has been shown to improve weight loss [27]. They continue to follow the reduced calorie diet until they achieve their prescribed weight loss goal, which is expected to occur for many by the end of the initial 8 week program. After reaching their weight loss goal, their calories are gradually increased to maintain this reduced weight level and a healthy, low calorie, low fat regimen is encouraged.

**Exercise** Large Changes participants are instructed to gradually increase their physical activity until achieving 250 minutes per week (5 days/week with 50 minutes per day) using activities similar in intensity to brisk walking and are encouraged to maintain this high level of activity over all subsequent years of the program.

**Self-monitoring** During the initial intervention program, during each refresher course, and if weight regain occurs, the Large Changes participants record their weight, diet (specific foods, portion sizes, and caloric and fat intake), and minutes of physical activity. These diaries are reviewed weekly by the interventionist, with written feedback provided. Participants are instructed to continue to record their weight daily throughout the trial.

**Maintenance** After completing the initial program, the primary emphasis is on daily self-weighing and using the weight data to determine if and when behavior changes are needed. Individuals who start to regain weight after the initial program, but do not exceed their baseline weight (i.e. in the “yellow” zone), are instructed to pay close attention to their diet and exercise (at current levels) and to problem solve and identify behavior changes that may be related to their weight gain. It is anticipated that there will be variability in the weight losses achieved by participants in the Large Changes group during the initial phase of the program, and thus differences in the amount of weight the person can regain before approaching their baseline weight. Thus, persons who lose more weight initially will have created a wider “yellow” or caution zone (i.e., a wider “buffer”) for themselves. If these participants enter the red zone, they are taught to return to self-monitoring with the reduced calorie intake goal of 1200–1500 or 1500–1800 calories per day. They are encouraged to use the structured meal plans and/or meal replacement products to achieve these goals. In addition, they are encouraged to make certain that they are reaching the 250 minute activity goal, and if needed, increase above this level. They are also encouraged to contact the program staff for red zone counseling session(s) to help them get back on track.

**Refresher courses** At each refresher course, participants are encouraged to create/maintain/or re-create a weight loss buffer of 5–10 pounds below baseline level. A calorie and physical activity prescription and daily self-monitoring are important aspects of the refresher. Individuals who have maintained their initial weight loss are allowed to lose more weight if they wish, provided that they do not reduce below a BMI of 18.5 kg/m^2^ (the lower end of the normal BMI range). The refresher classes offered to this group include cooking demonstrations and fitness activities to provide educational and interactive ways to encourage maintenance of the 5–10 pound weight gain buffer. Other campaigns formats are offered over time.

### Outcome measures

Assessments are completed at baseline, month 4, year 1, and year 2. Depending on when participants entered the trial, they may also complete a year 3 and year 4 assessment. All assessments are completed by staff members who are masked to the participants’ intervention assignment and have been certified by the Data Coordinating Center in the appropriate conduct of the measures. Specific protocols for the conduct of these measures are available from the Action for Health in Diabetes (Look AHEAD) trial [28]. Participants are provided a $50 honorarium for each assessment visit (with the exception of the baseline visit). Table 5 shows the schedule of assessments included in this trial.

**Table 5:** Data collection schedule for SNAP

**Measure**	**Month**					
	0	4	12	24	36	48
Anthropomorphic						
Weight (primary outcome)	X	X	X	X	X	X
Height	X	X	X	X	X	X
Waist circumference	X	X	X	X	X	X
Body composition with impedance	X	X	X	X	X	X
Body composition with BodPod (UNC only)	X	X	X	X	X	X
Behavioral and cognitive						
Diet (Food Frequency Questionnaire)	X	X		X		
Specific questions about diet	X	X	X	X	X	X
Physical activity: Paffenbarger	X	X	X	X	X	X
Sedentary activity	X	X	X	X	X	X
Objective (arm bands)	X	X	X	X		
Weight history	X					
Weight management strategies	X	X	X	X	X	X
Self-weighing	X	X	X	X	X	X
Eating disorders assessment	X	X	X	X	X	X
Eating inventory	X	X	X	X	X	X
Autonomous motivation	X	X	X	X	X	X
Smoking, alcohol use	X	X	X	X	X	X
Sleep habits	X	X	X	X	X	X
Neighborhood, environment	X	X	X	X	X	X
Medical						
Blood pressure	X	X	X	X	X	X
Fasting lipids, glucose, insulin	X			X		
Medication use	X	X	X	X	X	X
Medical events		X	X	X	X	X
DNA, serum, plasma	X			X		
Psychological assessments						
Depression (CES-D)	X	X	X	X	X	X
Life events	X	X	X	X	X	X
Perceived stress	X	X	X	X	X	X
Quality of life	X	X	X	X	X	X
Other questionnaires						
Demographic data	X	X	X	X	X	X
Contact information	X	X	X	X	X	X
Weight status of friends and family	X	X	X	X	X	X
Treatment preference, satisfaction and post-treatment feedback	X	X	X	X	X	X
Adherence (Intervention groups only)						
Attendance at adherence sessions		Throughout				
Monthly submission of weight data		Throughout				

#### Anthropometric and clinical measures

At each assessment visit, weight is measured in light clothes without shoes, on a calibrated scale, and height is determined using a wall mounted stadiometer. Two measures of each are completed and the average of the two is used. The weight and height measures are used to calculate Body Mass Index (weight in kilograms divided by height in meters squared). Waist circumference is measured at the midpoint between highest point of iliac crest and lowest point of costal margin using a Gulik tape measure; two measures of waist circumference are taken; if the difference exceeds 1.0 cm, a third measure is taken. Both sites complete measures of body composition with the RJL Systems Quantum II impedance machine. In addition, participants at UNC also have body composition assessed with the BodPod (COSMED USA, Inc.) Participants are asked to fast for 4 hours and to refrain from strenuous exercise for 8 hours prior to these body composition measures. Blood pressure is assessed with a Dinamap Monitor Pro 100. Cuff size is determined by arm circumference. Three readings are taken, with a 30-second wait between. At baseline and month 24 only, fasting blood samples are taken for analysis of lipids (total cholesterol, HDL-C, LDL-C and triglycerides) and glucose and insulin levels; DNA is collected for future genetic analyses in those who consent to this component. At each assessment, participants are asked to report all prescription and non-prescription medications and to indicate any health problems that they have experienced since the last assessment.

#### Behavioral measures

**Diet** Dietary intake is assessed at baseline, month 4 and month 24 with the Block Food Frequency, a semi-quantitiative food frequency questionnaire that has been used in a number of weight loss intervention trials including Look AHEAD, DPP, and PRIDE [29]. For each food item on the Food Frequency, participants report the frequency of consumption and the portion sizes consumed over the past month. An important reason for selecting this approach to dietary assessment is that the participants can complete the FFQ on-line at their own convenience. The FFQ is supplemented with specific questions related to frequency of meals at fast food restaurants, frequency of meals at other types of restaurants, and consumption of sweetened beverages.

**Physical activity** The Paffenbarger Activity Questionnaire (PAQ) [30] is administered as a measure of physical activity by trained interviewer at each assessment visit. The PAQ has been used to assess leisure time activity in many weight loss trials and can be scored to provide an estimate of calories expended per week in overall leisure time activity and in activities of light (5 kcal/min), medium (7.5 kcal/min), and high (10 kcal/min) intensity.

Given the increasing recognition of the importance of sedentary activity, independent of physical activity, sedentary activity is assessed at each assessment visit using a self-report questionnaire, which asks respondents to indicate the number of hours they spend on a typical weekday and a typical weekend day doing a variety of sedentary activities [31].

The SenseWear Pro Armbands (Body Media, Pittsburgh PA) provides an objective assessment of physical activity at baseline, month 4 and month 24 in order to confirm the self-report data and to determine whether participants in the small and large changes conditions have different patterns of activity that reflect the different exercise recommendations given to the two groups [32]. Participants are instructed to wear the device during all waking hours (except swimming and showering) for a full week; monitoring for at least 10 hours per day for at least 4 days in the week (including at least one weekday and one weekend day) is considered adequate for analysis.

**Weight management strategies** SNAP assesses both healthy (e.g. record what you eat daily, cut out between meal snacking) and unhealthy (e.g. take diet pills, fasting) weight control practices using questions compiled from Pound of Prevention [13], NHANES and the Weight Loss Maintenance trial. Participants are asked to indicate whether or not they used they have used each strategy within the past 4 months, and if so, to indicate how frequently they used the strategy. Participants also indicate whether they have participated in any other commercial weight loss programs including commercial and Internet programs and/or followed any other weight loss diets (e.g. Atkins). Frequency of self-weighing is assessed by asking participants how frequently they have weighed themselves within the past 4 month and year, ranging from several times a day, daily, a few times a week, weekly, once a month, or less than once a month to never.

**Other behaviors** Questionnaires are administered at baseline and at each assessment to assess smoking, alcohol intake, and sleep habits and any changes that occur over time.

#### Psychological measures

**Eating disorders assessment (EDA)** Participants complete a questionnaire used in Look AHEAD that assesses the frequency of binge eating episodes accompanied by loss of control, and the frequency of compensatory behaviors including vomiting, diuretics, and fasting. These data are used to exclude individuals with bulimia nervosa at baseline and identify any individuals who meet criteria for this eating disorder during the trial.

**Eating inventory** The Eating Inventory (TFEQ) [33] is a 51-item self-report instrument with three factors, assessing dietary restraint, disinhibition and hunger. The Restraint factor (range 0–21) assesses the degree of conscious control one is exerting over eating behaviors; the Disinhibition factor (range 0–16) measures susceptibility to loss of control over eating; and the hunger factor (range from 0–14) assesses hunger.

**Autonomous motivation** Autonomous motivation for preventing weight gain is measured with the Treatment Self Regulation Questionnaire [34,35]. Half of the items reflect autonomous motivation (e.g. “Because I feel that I want to take responsibility for my own health”) and half reflect controlled motivation (e.g. “Because I would feel guilty or ashamed of myself if I did not try to control my weight”).

**Depression** The Center for Epidemiologic Studies Depression Scale (CES-D) [36], a self-report depression scale designed to measure depression symptoms in the general population is administered at baseline and each assessment visit.

**Life events** The life events questionnaire from the Coronary Artery Risk Development in Young (CARDIA) study lists 67 events and participants are asked to indicate whether or not that event has occurred since the last visit [37]. We have chosen to use the CARDIA life events questionnaire in preference to other similar questionnaires (e.g. Holmes and Rahe) [38] because it was developed specifically for young adults and reflects the type of life events that occur most commonly in this age group.

**Perceived stress** The Cohen Perceived Stress Scale [39] is a 4-item self-report instrument that captures the participant’s perception of stress in their lives over the past month. The Perceived Stress Scale poses general questions about current stress levels.

**Quality of life** All participants complete the CDC Health-Related Quality of Life measure (commonly referred to as “Healthy Days Measures”) at each assessment [40]. This 4 item questionnaire has been utilized in the BRFSS and NHANES and has been shown to have appropriate reliability, validity, and responsiveness to change.

#### Supporting measures

Basic demographic information are collected, including age, race/ethnicity, occupation, education and prior experience in weight loss programs.

**Weight status of friends and family** Based on the increasing recognition of the importance of social networks, participants are asked to indicate the weight status of their friends and family members. This information is collected at each assessment visit to determine if changes in weight in the participant are related to the weight status of others in their social network.

**Treatment preference and satisfaction** Prior to randomization, each participants’ preference for each of the three arms of the trial is recorded to determine whether initial preference for small or large changes group relates to outcome in participants assigned to their preferred or non-preferred alternative. Participants also complete a post-treatment process evaluation common to the EARLY studies to assess satisfaction with outcome and program components.

**Measures of adherence** Data are collected on the number of intervention visits attended in the two active intervention groups, including any individual make-up sessions attended and individual “red-zone” counseling sessions. Data are also available on the percent of weeks in which participants reported their weight on the study website, mobile web, via SMS or email.

**Measures of intervention fidelity** To ensure that the interventions delivered to the Small Changes and Large Changes groups remain distinct from each other and appropriately reflect the intervention protocol, all intervention sessions are audio taped. A random 20% of these sessions are reviewed by an interventionist or investigator who did not conduct the intervention sessions and is masked as to which group is being conducted. The reviewer listens to the session tape and completes a fidelity checklist that was developed specifically for this trial to ensure minimal contamination between intervention groups and confirm that the essential content for each session is being covered.

### Safety, and monitoring of serious adverse events and other medical events

Data are collected at in-person visits and if contacted by a participant between visits regarding any medical issues that might affect safety of the intervention or outcomes (e.g. pregnancy). Serious adverse events are defined as hospitalizations, fatal or life-threatening events, events resulting in significant or persistent disabilities, birth defects or congenital abnormalities, or important medical events that investigators judge to represent significant hazards or harm to research subjects. Other medical events and alert values for assessments of clinical relevance are also monitored and reported to participants. Standard guidelines are used to determine alert values and recommend medical follow-up to participants for blood pressure, lipid and glucose levels. If a participant has a greater than 20% weight loss from baseline, reaches a BMI ≤ 18.5 kg/m^2^ or loses more than 15 pounds in a month, the participant is seen and counseled. If further weight loss occurs, intervention activities are suspended. If a participant develops an eating disorder, the participant is referred to care, and intervention is discontinued and not resumed until cleared by their care provider. Intervention activities are also stopped during pregnancy or if there are injuries or illnesses where weight loss and/or physical activity would be contra-indicated. Intervention is resumed 6-months post-partum or when injuries or illnesses resolve.

### Procedures for minimizing dropouts and improving retention

A systematic protocol is followed to minimize dropouts. Participants in the intervention are called or sent an email reminder before each session. If a participant has an unexpected absence, they are contacted and helped to solve any barriers to attendance. The session materials are emailed or sent to those who do not attend and a make-up session is offered. Top priority is for assessment visits. At baseline, names and addresses of several friends and family members who can be contacted are obtained for use if we lose touch with the participant. Birthday cards, holiday cards an annual token gift, and periodic newsletters are also to improve retention. The biggest source of dropouts in prior studies with this age group is pregnancy (15% of women in Health Hunters [41] had a pregnancy; 11% of the women in POP [13]). We stay in touch with women during pregnancy and allow them to return to the study at 6 months post-partum; we continue to include their weight data in our analyses except during their actual pregnancy and the 6-months post-partum.

### Sample size calculation

The targeted sample size (N=600 total; N=200/arm) is projected to provide ≥ 90% statistical power to detect an average pairwise difference of 3.0 lbs (1.36 kg) weight change over time between intervention arms, while accommodating anticipated rates of lost follow-up (an accumulating 7.5% at Month 4, an additional 7.5% at Month 12, and 5%/year thereafter). Comparisons of this primary outcome will be based on generalized linear models for longitudinal data. The targeted sample size provides 90% power to detect a relative 28% reduction in the proportion of participants who gain weight over time, an important secondary outcome, based on generalized estimating equations.

### Analysis plan

The primary outcome measure, changes in weight from baseline over time, is assessed at 4-, 12-, 24-, 36- (80% of cohort), and 48-months (20% of cohort). All measured weights will be included in the analyses, except any that may have occurred during a pregnancy or within 6 months post-partum. Weight changes will be contrasted among intervention groups using generalized linear models fitted by maximum likelihood with an unstructured covariance matrix [42], with clinic site included as a covariate. Estimated mean differences for each pairwise comparison will be developed using linear contrasts and assessed with Wald statistics, using Bonferroni adjustment to control total Type I error to be 0.05 across the three comparisons.

In the primary analysis, missing weight measurements will be assumed missing at random, but additional analyses will be conducted related to missing data (e.g. comparing baseline characteristics of completers vs non-completers and creating propensity scores). If missing data appear to have the potential of influencing interpretations, we will also conduct multiple imputations using several different models for non-ignorable missingness. These will be used to assess the range of impact that missing data may exert on our results. To gauge the sensitivity of our results to any changes in height, we will conduct supporting analyses of changes in BMI.

The secondary aims will be examined as follows. Pairwise differences in the secondary outcome measure, weight gain of greater than 1 pound from baseline (yes/no), will be assessed using generalized estimating equation (GEE) methods. Pairwise differences in mean weight changes from baseline to 24 months post-randomization among intervention arms will be assessed with linear contrasts and Wald tests within the framework of the general linear models used for the primary aim. Two-sided tests will be used with Bonferroni-adjustment to maintain overall Type I error for this secondary aim at 0.05. Generalized linear models will be used to compare the changes in behaviors and risk factors over time among the three treatments in a manner similar to that used for assessing weight changes. The effects of demographic and psychological measures on the outcomes will be examined by including initial BMI, ethnicity, age, scores on the eating inventory, and intervention preference. Significant interaction effects will be plotted to illustrate the moderating effects, further assisting the interpretation for whom and under what circumstances the intervention has different effects. To examine potential mediators of the effect of intervention, i.e., whether or not there is evidence that changes in diet, physical activity, restraint, and self-regulatory behaviors may be in the causal pathway between the intervention and weight gain, a series of three linear regression models will be fitted to test for mediators following the procedures introduced by Baron and Kenny [43]. The Sobel’s test will be used to test the significance of the indirect effect. The joint effects of multiple mediators will also be tested [44]. For the secondary outcome of weight gain (yes/no), logistic regression models will be fitted to assess meditational effects [45].

We pre-specify three planned subgroup comparisons. We will assess, using tests of interaction, whether the relative efficacy of the intervention varies according to baseline BMI (<25 kg/m^2^ versus ≥25 kg/m^2^), age (<25 years versus ≥25 years), and gender.

The fidelity of intervention delivery within the two clinical sites will be assessed by comparing measures of adherence and weight control. Adverse events will be tallied by intervention assignment and for important clinical subgroups. We will report event rates per person years and use Poisson regression to compare rates of any commonly occurring events among intervention groups.

### Data and safety monitoring board (DSMB)

NHLBI has constituted a DSMB to oversee the progress and safety of this trial, with members providing expertise in behavioral weight control, epidemiology, biostatistics, and cardiovascular disease. This Board meets approximately twice/year, reviews recruitment and retention, adverse events, and other potential safety concerns weight loss outcomes, and overall study conduct.

## Discussion

To date, only a few large-scale randomized clinical trials have targeted weight gain prevention, and their success has been limited. In addition, young adults, the age group at greatest risk for weight gain, have been underrepresented in these studies. The SNAP randomized controlled trial is designed to test a self-regulation based approach to weight gain prevention in normal weight and overweight individuals 18–35 years of age. Participants are recruited from two demographically distinct regions – the Greater Providence area and Raleigh-Durham-Chapel Hill, NC, with efforts made to ensure gender, race and ethnic variability within the sample. Two different types of messages within a self-regulation framework are compared – one emphasizing small daily changes in energy intake and expenditure (Small Changes) and the other advocating the creation of a weight loss buffer to protect against anticipated weight gain (Large Changes). We hypothesize that both approaches will be more effective than a self-guided control group in preventing weight gain over an average 3 year follow-up, with the best outcomes predicted in the Large Changes group.

While making small changes in diet and physical activity has been advanced in the popular press as a means of weight management, limited empirical evidence is available to support this public health message and recent papers raise conceptual concerns about this approach [46]. With its extended follow-up period and large sample size, SNAP will contribute significantly to the literature about the viability of this approach. If found to be efficacious, the notion that making one or two small changes everyday can result in weight gain prevention over several years might have widespread appeal. The large changes approach has more empirical support due to its success in the Women’s Healthy Lifestyle Project and secondary analyses from the Pound of Prevention trial. SNAP will provide evidence as to whether this approach works for young adults, a developmental period with its own unique challenges including establishing autonomy, adjusting to school-work transitions, and navigating interpersonal relationships and child rearing.

Both interventions are designed with dissemination in mind with limited face-to-face interaction and utilization of internet, email, and smart phone communication to engage participants in the treatment process. If either or both of the large changes and small changes approaches are successful in preventing weight gain, an important next step will be to translate the interventions beyond the research setting and into community or commercial use.

## Competing interests

The authors have indicated that they have no competing interests.

## Authors’ contributions

RW was responsible for study design and the grant submission and oversees the study at The Miriam Hospital; DT collaborated on study design and grant submission and oversees the study at University of North Carolina; ME collaborated on study design and grant submission, and oversees the Coordinating Center at Wake Forest School of Medicine, AG collaborated on development the interventions and assessment tools; JL collaborated on development of the intervention and fidelity monitoring and pilot tested the interventions; EFR coordinates the project at The Miriam Hospital; KE coordinates the project at UNC, LP coordinates the project at Wake Forest, JB oversees the development of forms and monitoring of data; CL oversees all medical and safety aspects of the trial. All authors helped in the development of the manuscript and reviewed its content. All authors read and approved the final manuscript.

## Pre-publication history

The pre-publication history for this paper can be accessed here:

http://www.biomedcentral.com/1471-2458/13/300/prepub
